# Preventing new substance use behaviors in youth: evaluation of a two-year comprehensive program

**DOI:** 10.3389/fpsyg.2024.1339751

**Published:** 2024-07-05

**Authors:** Oulmann Zerhouni, Sandra Loisy, Renaud Bouthier, Valentin Flaudias

**Affiliations:** ^1^Laboratoire Parisien de Psychologie Sociale, Département de Psychologie, Université Paris Nanterre, Nanterre, France; ^2^Centre de Recherche sur les Fonctionnements et Dysfonctionnements Psychologiques (CRFDP, EA 7475), University of Rouen Normandy, Mont-Saint-Aignan, France; ^3^Association Avenir Santé, Lyon, France; ^4^Laboratoire de Psychologie des Pays de la Loire (LPPL UR 4638), Nantes Université, Nantes, France; ^5^Pôle Psychiatrie B, CHU Clermont-Ferrand, Clermont-Ferrand, France

**Keywords:** prevention, ecstasy, cannabidiol, nitrous oxide, field interventions

## Abstract

**Introduction:**

The Avenir Santé Association implemented a comprehensive prevention program targeting the consumption of the emerging psychoactive substances ecstasy (MDMA), cannabidiol (CBD), and nitrous oxide (N_2_O).

**Methods:**

The program was evaluated through four actions: (i) training for association workers (*n* = 84) (ii) on-site student party interventions (*n* = 248), (iii) social network-based prevention (*n* = 186), and (iv) provision of prevention tools for party organizers (*n* = 148).

**Results:**

Results showed a significant increase in understanding of emerging substances among association workers, with a pre-training score of *M* = 15.76 (SD = 3.65) and a post-training score of *M* = 18.29 (SD = 2.50). Increased awareness and reflective attitudes toward substance use were observed among young people participating in field actions, with pre- and post-intervention scores for MDMA use intentions being *M* = 15.89 (SD = 4.60) and *M* = 19.17 (SD = 3.33), respectively. Similarly, awareness of CBD effects increased from *M* = 14.18 (SD = 4.14) to *M* = 17.60 (SD = 3.31). Exposure to Instagram posts on N_2_O led to more negative attitudes toward N_2_O among young people, with a significant change in scores from *M* = 8.16 (SD = 1.57) to *M* = 8.42 (SD = 1.26). However, exposure to a website providing information about emerging substances did not produce any significant effect.

**Discussion:**

In conclusion, this initiative underscores the usefulness of facilitator training, field interventions, and certain online information strategies for substance judgment and usage intentions. Future prevention programs can advantageously incorporate these actions.

## Introduction

The 2022 ESCAPAD survey (Enquête sur la santé et les comportements lors de la préparation à la défense) found a decrease in the use of substances such as tobacco, alcohol and cannabis among the French compared to 2000 (OFDT, 2023). For example, Experimentation with tobacco (at least once in a lifetime) fell from 77.6 percent to 45.5 percent; with alcohol from 94.6 percent to 80.6 percent; and with cannabis from 45.6 percent to 29.9 percent. In contrast, the use of newer substances, including ecstasy (MDMA), despite its longstanding use, has exhibited a notable rise in recent times. Similarly noteworthy are the increases observed in the use of nitrous oxide (N_2_O) and cannabidiol (CBD) (Wheeler et al., 2020; Ponce-Blandón et al., 2021; Ferreira et al., 2022; Inquimbert et al., 2022; Cohen et al., 2023). This increased usage has been accompanied by normalization of substance use among students, with fewer viewing such substance use as problematic (van den Bos et al., 2022). Long-term effects of MDMA, with reduced attention control (Peroutka, 1989; Vollenweider et al., 2002; Cole and Sumnall, 2003; Parrott, 2005; Piper, 2007), and of N_2_O, with the development of neuropathies, are well-known (Lundin et al., 2019; Redmond et al., 2022). However, the negative impacts of long-term CBD use remain largely unknown, although some research points to potential effects on attention and vigilance, especially in psychiatric patients (Millar et al., 2019; Batalla et al., 2021; Rudisill et al., 2023). The scientific literature has highlighted several criteria for the effectiveness of a preventive action [see for example (Kempf et al., 2017)]. First, it is important to act on multiple levers rather than a single action. Indeed, one of the most influential explanatory models of substance use in psychology, the theory of planned behavior (Ajzen, 1991), shows that behavioral motivation is influenced by, among other things, peers, environmental social norms, and the individual’s own representations. These different interactions highlight the importance of interventions that target these different sources of behavioral influence [see (Kempf et al., 2017) for more details on the different models]. It is also important to train the professionals who are in contact with the students concerned in order to ensure a change in social norms in the students’ immediate environment. In the same spirit, it is important to be able to carry out the action in places where students are present (in particular social networks or party venues). Avenir Santé Association has addressed these trends with a preventive program for 18–25-year-olds, encompassing staff training, outreach initiatives in student party venues, prevention awareness via social media, and provision of prevention tools for party organizers (Flaudias et al., 2015; Kempf et al., 2017). The purpose was to bolster knowledge and self-efficacy to instigate preventive actions, and to evaluate the impact of these actions in different environments.

This prevention project was designed to engage young individuals and key influencers within their environments to reduce the use of emerging psychoactive substances such as MDMA, CBD, and N_2_O. The project’s objective was to enhance knowledge, self-efficacy, and awareness of substance use among specific target groups, including workers, adolescents, social media users, and event organizers. Through both direct and indirect engagement strategies, the initiative sought to encourage the adoption of informed and preventative behaviors among these communities.

This strategy included both direct contact with the target group of young people and indirect engagement with the professionals that students might encounter during this period of their lives. The project was divided into four separate preventive actions.

- *Action 1—Training intervention specialists*: The first step was to equip a group of professionals with the knowledge and skills to disseminate awareness and prevention messages effectively. Training emphasized a comprehensive understanding of substance misuse and its consequences, forceful communication skills, and insight into the psychological and behavioral repercussions of substance use.
- *Action 2—Direct actions with youth through in-person outreach*: The next phase involved direct interaction with youth at their common gathering spots. Outreach teams, separate from the trained individuals in *Action 1*, initiated face-to-face engagements to give personalized advice, correct misconceptions, and build rapport.
- *Action 3—Social media awareness campaign*: To leverage the widespread influence of social media among youth, the project used digital platforms (e.g., Instagram) for educational messaging about psychoactive substance risks. This approach utilized the convenience and broad reach of social media, creating an interactive space for discussion and learning.
- *Action 4—Web support for party organizers*: The project aimed to inform actors creating potential substance use environments by providing a dedicated webpage for party organizers. This resource offered vital information about psychoactive substances, tips on identifying misuse signs, emergency handling, and overall attendee safety, thus expanding the network of informed individuals to help reduce substance misuse.

## Methods

### Participants and procedure

Each action was evaluated separately.

*Action 1* (*Training intervention specialists*) participants in *Action 1* had to be volunteers from the Avenir Santé association who had attended the specialized training course on new psychoactive substances. They must have given informed consent to participate and completed both the pre- and post-training questionnaires. Exclusion criteria were those who did not give informed consent, or those who did not complete both questionnaires. Eighty-four volunteers from the Avenir Santé Association attended a specialized training course on emergent psychoactive substances and prevention of their use. Participants were asked to complete a questionnaire twice—once before the beginning of the training (pre-training) and once after the conclusion of the training (post-training). Number of correct responses was taken as the primary measure of the training’s effectiveness. The materials for evaluating knowledge related to emerging substances included a specially designed questionnaire, tailored to assess the impact of the training on knowledge about emerging substances. It comprised three main sections, each addressing one substance: nitrous oxide (7 items), MDMA (7 items), and CBD (6 items) (see Appendix A for more details). Each section comprised multiple-choice questions that probed the respondents’ understanding of the effects of the substance (e.g., “What effects do consumers look for?”), how it is consumed (e.g., “What are the different ways it is consumed?”), and viable strategies for reducing its use (e.g., “What risk and harm reduction advice could you give?”).

*Action 2* (*Direct actions with youth through in-person outreach*) aimed to assess the impact of interactions with Avenir Santé Association outreach teams on participants’ knowledge about the effects of different substances and their intentions to reduce their use. Inclusion criteria for *Action 2* required participants to be young adults above the age of 18 who were present at student party venues where Avenir Santé Association conducted outreach interventions. They had to provide informed consent and participate in the outreach sessions. Participants were excluded if they were not in the target age range, did not provide consent, or were not present at the intervention sites. The evaluation aimed to compare the effects of outreach interventions focused on emerging substances (N_2_O, MDMA, CBD) with those focused on more familiar substances (alcohol and tobacco). (See Appendix B for questionnaires on attitudes and intentions). Each member of the outreach team discussed the use of one or more emerging substance (MDMA, CBD, N_2_O) or alcohol and tobacco with the people they approached. Depending on the substance for which the outreach was conducted, the respondent completed the appropriate questions on attitudes and use intentions. Volunteers from the “Avenir Santé Association” gave participants at festive events a form presenting the risks associated with consuming one or more emerging substance and/or alcohol and tobacco. Sociodemographic data (age, sex, place of residence) were also collected. The total sample consisted of 248 persons from various regions in France (mean age 21.9 years, SD = 3.86, 48.6% female).

*Action 3* (*Raising awareness through social media*) focused on assessing the impact of Instagram posts from an online prevention campaign. We evaluated Instagram posts that formed part of a real prevention campaign (there were 672,797 interactions with the posts concerning N_2_O, summing views, likes, and shares) on the consumption of (i) alcohol or (ii) N_2_O regarding their impact on attitudes and intentions to consume them among students at two French Universities. Since it was difficult to evaluate the target group directly without jeopardizing the effectiveness of the intervention, we proposed an evaluation of a group of students who corresponded to the target group (age and diversity of profiles). Participants in *Action 3* interacted with Instagram posts as part of an online prevention campaign and were young adults, who were students at the Universities of Nantes and Nanterre. Inclusion required informed consent and participation in the online study. Exclusion criteria included being outside the age range or not giving consent. Participants took part in an online study in which they were asked to report their attitudes and intentions to consume alcohol and N_2_O. Each participant was randomly assigned to an experimental condition where they viewed either (i) six posts on N_2_O (N_2_O exposure condition) or (ii) six posts on alcohol (alcohol-exposure condition). In both conditions, we measured participants’ attitudes and consumption intentions before and after they were exposed to the Instagram posts. The attitudes were evaluated on four scales that measured perceptions of healthiness (from 1: unhealthy to 9: healthy), wisdom (from 1: unwise to 9: wise), goodness (from 1: bad to 9: good), and safety (from 1: unsafe to 9: safe) associated with the substances. Additionally, participants were asked whether they had previously consumed alcohol or N_2_O. Demographic information including age and sex was also collected. We also measured the quantity of information that the exposed individuals correctly memorized to ensure that the participants had actually viewed the images (see Appendix C for all Instagram Posts used and questions on attitudes and intentions). The sample comprised 186 psychology students from the Universities of Nantes and Nanterre (mean age 20 years, SD = 3.4; female = 77.42%).

In *Action 4* (*Supporting party organizers through a dedicated web page*), we conducted an online experiment to assess the impact of a website^1^ offering information about (i) alcohol, and (ii) emerging addictive substances (MDMA, N_2_O, CBD). *Action 4* included young adults aged 18 to 25 from the universities of Nantes and Nanterre who visited the montetasoiree.com website. Participants had to give informed consent and complete the pre- and post-exposure questionnaires. Exclusion criteria were those outside the specified age range, those who did not give their consent. The goal was to determine whether exposure to these webpages would affect attitudes toward, and intentions to consume, alcohol and emerging substances. Like in *Action 3*, the website was taken from a pre-existing site that had been used for an alcohol and emerging substances prevention campaign. As in *Action 3*, it was complicated to evaluate the target population directly without compromising the effectiveness of the intervention, so we proposed an evaluation of a group of students who corresponded to the target (age and diversity of profiles). A total of 66 students were exposed to the page containing information on alcohol, while 64 students viewed the page on emerging substances (mean age 19.68 years, SD = 3.6; 94% female) from the Universities of Nantes and Nanterre. The sex ratio indicated a much larger proportion of female participants, which could potentially affect the results given that there may be sex differences in attitudes and behaviors related to alcohol and drug use. The participants were asked to respond to an online questionnaire before and after viewing a webpage. The webpage they viewed was randomly selected between (i) a page from the website providing information on alcohol drinking (i.e., effects on health and how to drink responsibly), or (ii) a page discussing emerging substances (i.e., effects on health and how to mitigate their effect when consuming). We measured (i) attitudes toward alcohol and emerging substances, (ii) intentions to consume alcohol and emerging substances, and (iii) age and sex.

The evaluation was conducted according to the fundamental principles of the Declaration of Helsinki. Authorizations were obtained by the department of psychology, by the institutions where the study was conducted and by the ethical committee (Authorization No. 30112023). Participants were informed of the conditions, context, and characteristics of the evaluation, their rights, and the right to withdraw whenever they decided. They were not paid to participate in the evaluation.

### Data analysis strategy

For *Action 1*, correct answers before and after the training were compared using a repeated measures ANOVA. The objective was to test whether (i) the training actually improved the participants’ knowledge about emerging substances, and (ii) whether information about certain substances was better before training and better retained than information about other substances. We used the time of measurement (before or after training), the substance (N_2_O, MDMA, CBD), and the interaction between these two variables as predictors. All descriptive statistics for *Action 1* are available in Table 1.

**Table 1:** Descriptive statistics for exam score before (pre) and after (post) training.

	*N*	Mean	Median	SD
Score CBD pre	96	15.760	16.000	3.650
Score PA pre	96	14.177	15.000	4.142
Score MDMA pre	96	15.885	16.000	4.599
Score CBD post	84	18.286	18.000	2.496
Score PA post	84	17.595	17.000	3.312
Score MDMA post	84	19.167	20.000	3.325

For *Action 2*, two mixed linear models were run to evaluate the impact of the substance on which information was given, the location of the outreach, and the knowledge about the possibilities of managing substance use and knowledge about the effects of the substances on (i) future intentions to use emerging substances and (ii) future intentions to consume alcohol and tobacco. The regions where data were collected was entered as a random intercept in the models to control for potential variation across regions. All descriptive statistics for *Action 2* are available in Table 2.

**Table 2:** Descriptive statistics for *Action 2*.

	Sex	*N*	Mean	SD
Do you know the long term effect on health of emerging substances?	F	129	6.895	3.034
	M	94	6.750	2.599
Do you have the intention of consuming emerging substances in the future?	F	115	5.017	4.335
	M	87	4.253	4.300
Souhait diminuer conso émergentes	F	25	3.960	3.116
	M	14	2.071	2.674
Conséquences conso émergente sur la santé	F	24	5.125	3.395
	M	13	5.077	3.730
Knowing the effect of drinking and smoking on health	F	128	7.461	2.421
	M	99	6.924	2.127
Intention to diminish drinking and smoking	F	125	3.772	3.549
	M	99	4.404	3.722
Wishing to diminish drinking and smoking	F	22	5.136	3.992
	M	21	5.762	3.390
Consequences of drinking and smoking	F	22	5.568	4.112
	M	23	4.913	3.554
Age	F	136	21.485	3.428
	M	109	22.514	4.311

For *Action 3*, four general linear models were used to evaluate the impact of the type of posts participants were exposed to (alcohol vs. N_2_O condition), memorized information, interaction between the type of post and memorization scores, age, and sex on attitudes and intentions toward alcohol and N_2_O. All descriptive statistics for *Action 3* are available in Table 3.

**Table 3:** Descriptive statistics per type of exposure for *Action 3*.

	Type of exposure	*N*	Mean	SD
Age	N_2_O exposure	81	20.605	4.300
	Alcohol exposure	71	19.451	1.970
Attitudes toward N_2_O before exposure	N_2_O exposure	87	8.158	1.565
	Alcohol exposure	72	8.208	1.552
Attitudes toward alcohol before exposure	Alcohol exposure	87	6.560	1.918
	Alcohol exposure	72	6.380	2.021
Attitudes toward N_2_O after exposure	N_2_O exposure	82	8.424	1.256
	Alcohol exposure	72	8.174	1.532
Attitudes toward alcohol after exposure	N_2_O exposure	82	6.490	1.812
	Alcohol exposure	72	6.663	1.990
Intention to consume N_2_O before exposure	N_2_O exposure	40	2.950	8.391
	Alcohol exposure	45	2.022	9.016
Intention to consume alcohol before exposure	N_2_O exposure	69	55.304	35.843
	Alcohol exposure	63	55.397	37.514
Intention to consume N_2_O after exposure	N_2_O exposure	43	3.372	10.497
	Alcohol exposure	40	2.200	9.495
Intention to consume alcohol after exposure	N_2_O exposure	67	54.731	33.932
	Alcohol exposure	62	54.694	36.647

For *Action 4*, four repeated measures analyses of variance (ANOVA) were conducted to assess the impact of the type of information (alcohol vs. emerging substances) on attitudes and future consumption intentions for alcohol and emerging substances. The threshold of significance was set at 0.05 The significance threshold was set at 0.05 and a Bonferroni correction for multiple tests was applied for *Action 1*, lowering the threshold to 0.001 for this action. All analyses were performed using the JAMOVI software version 2.3.24. All descriptive statistics for *Action 1* are available in Table 4.

**Table 4:** Descriptive statistics per type of substance on the website for *Action 4*.

	Substance on the website	*N*	Mean	SD
Pre-attitudes toward PA	N_2_O	73	7.925	1.678
	Alcohol	75	7.963	1.759
Pre-attitudes toward alcohol	N_2_O	76	6.454	2.007
	Alcohol	76	6.421	1.851
Post-attitudes toward alcohol	N_2_O	69	6.659	1.981
	Alcohol	66	6.943	1.867
Post-attitudes toward PA	N_2_O	64	8.438	1.121
	Alcohol	68	8.353	1.234
Age	N_2_O	68	19.721	3.124
	Alcohol	70	211.214	1602.729

## Results

**Figure 1:**
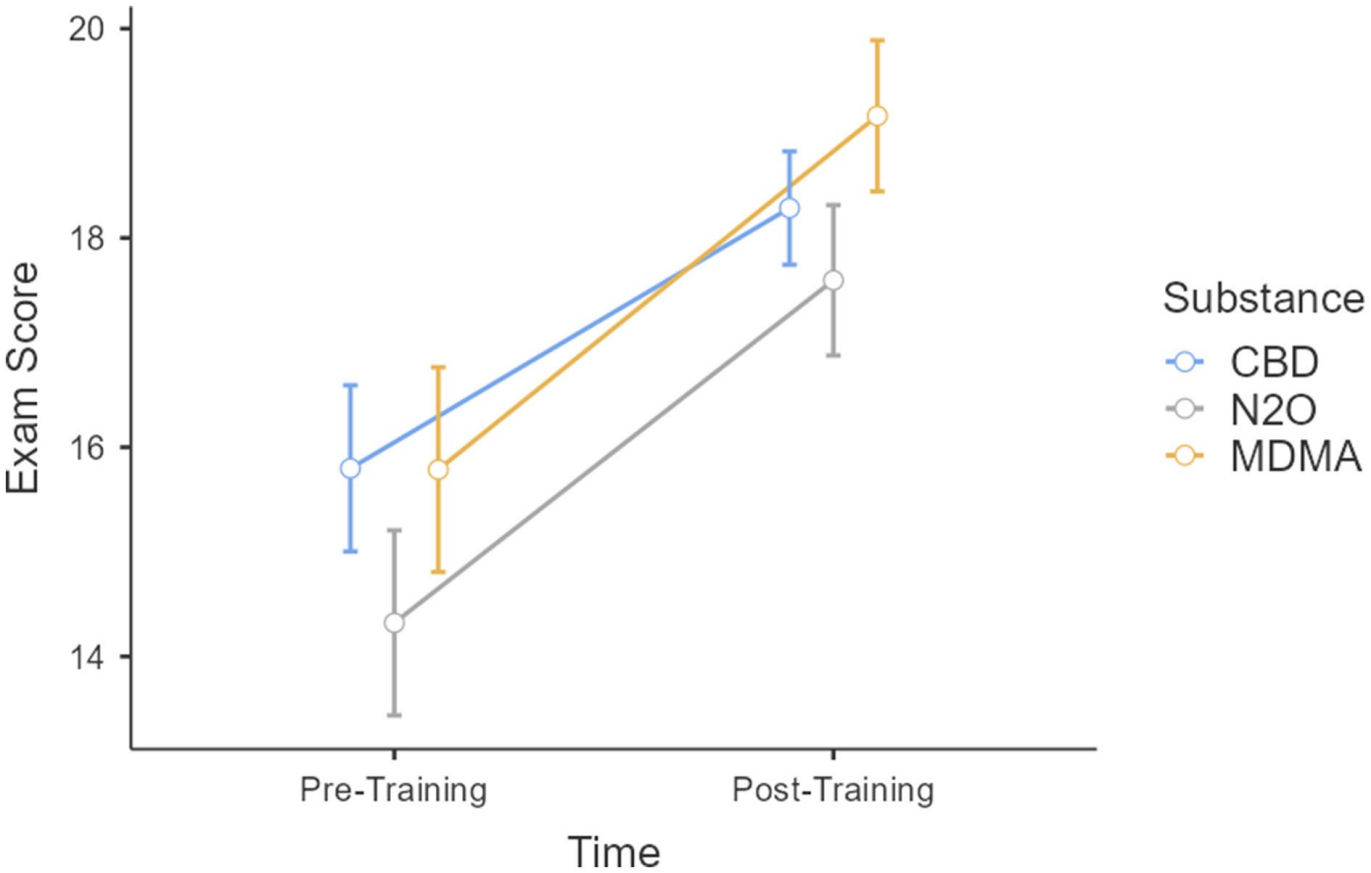
Score as a function of the time when completed (before and after training), and according to the substance involved (CBD, MDMA, N_2_O).

Results for *Action 1*, showed a significant difference in post-training answer across different substances [*F*(2,166) = 16.28, *p* < 0.001, *η*_p_^2^ = 0.16], and a higher proportion of correct answers after training than before training [*F*(1,83) = 37.36, *p* < 0.001, *η*_p_^2^ = 0.31]. However, no interaction between substance and time of measurement was observed [*F*(2,166) = 1.91, *p* = 0.152, *η*_p_^2^ = 0.02], suggesting that training had a similar impact on all substances. Specific comparisons before and after training for each substance indicated a significant difference for CBD [*t*(83) = −4.87, *p* < 0.001], N_2_O [*t*(83) = −3.01, *p* = 0.039], and MDMA [*t*(83) = −6.14, *p* < 0.001, see Figure 1].

Results for *Action 2* on effects on intentions to reduce their consumption regarding alcohol and tobacco, showed that sex emerged as a significant predictor. Males reported significantly higher intentions to reduce their consumption than females [*β* = 0.948, *t*(213.026) = 2.015, *p* = 0.045]. Age was also a significant predictor, with older participants reporting stronger intentions [*β* = 0.140, *t*(210,483) = 2.199, *p* = 0.029]. Participants informed about alcohol reported significantly lower intentions to drink [*β* = −1.061, *t*(213.986) = −2.167, *p* = 0.031]. However, amount of previous consumption was not statistically significant, although it showed a trend in which those who had previously consumed reported a higher intention to consume again [*β* = 1.360, *t*(213.387) = 1.894, *p* = 0.060]. Individuals with greater knowledge of the health effects of tobacco and alcohol reported higher intentions to reduce their consumption [*β* = 0.232, *t*(212.323) = 2.369, *p* = 0.019]. Concerning random effects, the variance associated with the intercept for the region was 2.053 with a standard deviation (SD) of 1.433 and an intraclass correlation coefficient (ICC) of 0.169. The residual variance was estimated to be 10.118 with an SD of 3.181. Results on effects on intentions to reduce their consumption regarding emerging substances showed that a total of 246 participants were evaluated (mean age = 21.9 years, SD = 3.8; 55.51% female). We found a marginally significant trend where participants informed about the effects of N_2_O showed only slightly increased intentions to reduce consumption [*β* = 0.932, *t*(190.386) = 1.792, *p* = 0.075]. We also found that participants with a history of substance use reported lower intentions to reduce their consumption [*β* = 2.414, *t*(192.945) = 3.879, *p* < 0.001]. Moreover, greater awareness was found to be associated with a small but significant increase in intentions to decrease substance use [*β* = 0.253, *t*(189.375) = 3.011, *p* = 0.003]. However, there was no significant effect of having been informed about other specific emerging substances (MDMA, CBD). The random effects analysis showed a standard deviation of the intercept across regions of 2.541, giving a variance of 6.458. This variability in the intercept corresponds to an intraclass correlation coefficient (ICC) of 0.413, suggesting that approximately 41.3% of the variability in intentions was due to differences between the regions. The residual standard deviation was 3.032 with a variance of 9.191.

**Figure 2:**
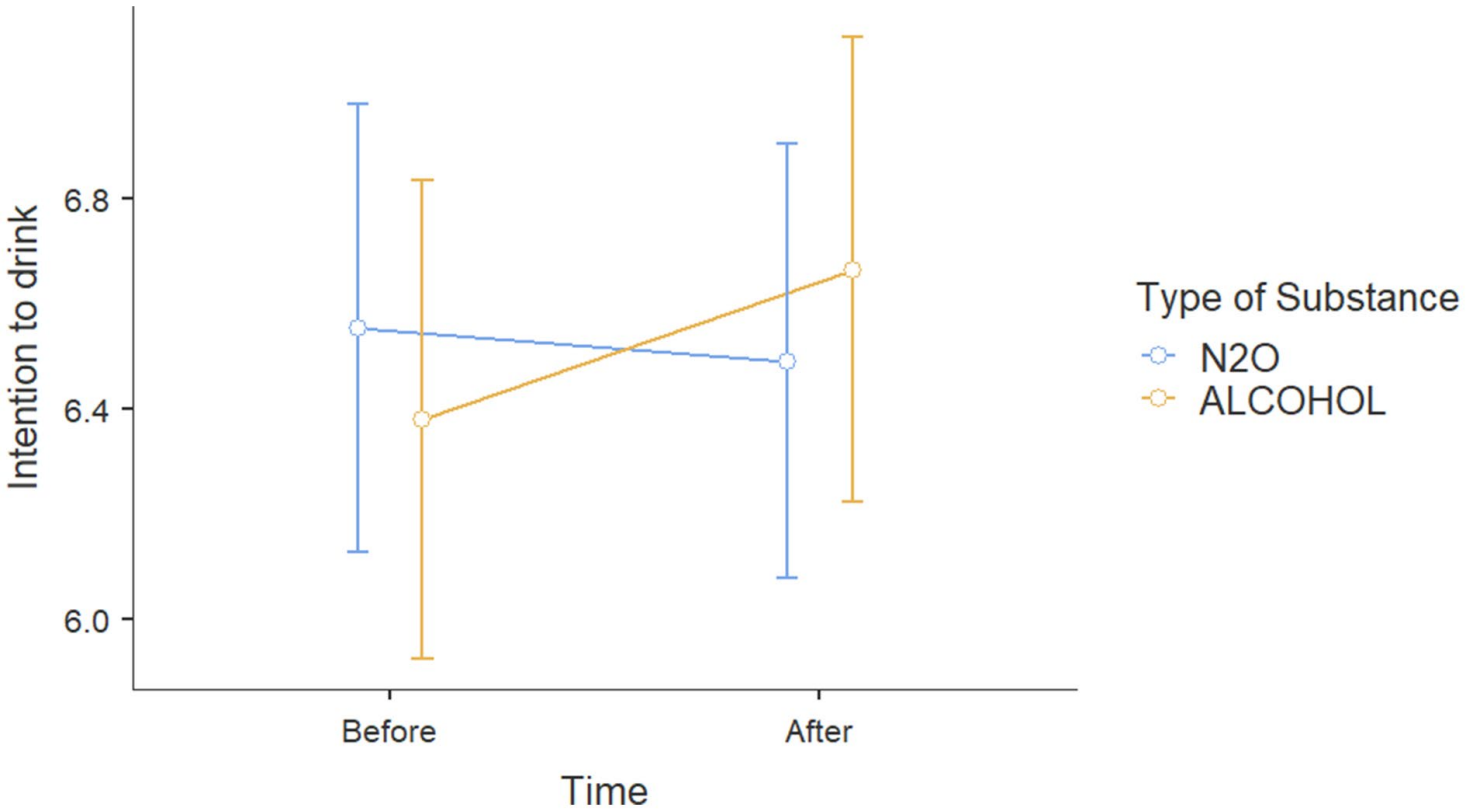
Scores at attitudes toward alcohol before and after viewing Instagram posts about either alcohol or N_2_O.

Results for *Action 3*, showed that on effects on attitudes toward alcohol for participants with alcohol prevention posts, there was no significant age effect on attitudes toward alcohol [*β* = 0.008, *t*(145) = 0.429, *p* = 0.669], nor any sex effect [*β* = 0.151, *t*(145) = 0.615, *p* = 0.53]. Similarly, the proportion of correct responses to the questionnaire on the content of the posts showed no association with attitudes toward alcohol [*β* = 0.004, *t*(145) = 0.059, *p* = 0.953]. However, participants in the alcohol-exposure condition showed more negative attitudes toward alcohol [*β* = 0.342, *t*(145) = 2.534, *p* = 0.012]. This impact was not moderated by the number of correct responses to the alcohol questionnaire [*t*(145) = 0.181, *p* = 0.857] (Figure 2).

**Figure 3:**
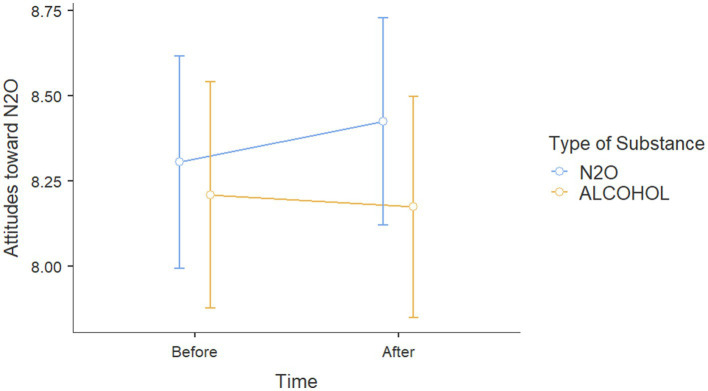
Attitude scores toward N_2_O before and after viewing Instagram posts about either alcohol or N_2_O.

These results also showed on effects on attitudes toward nitrous oxide for participants with nitrous oxide prevention posts that the impact of exposure to N_2_O prevention posts was found to increase with the number of correct responses to the N_2_O questionnaire [*β* = −0.332, *t*(145) = −2.016, *p* = 0.046, *η*_p_^2^ = 0.027]. This suggests that participants exposed to N_2_O information on social networks demonstrated more negative attitudes toward this substance when they achieved higher scores on the N_2_O questionnaire (Figure 3).

**Figure 4:**
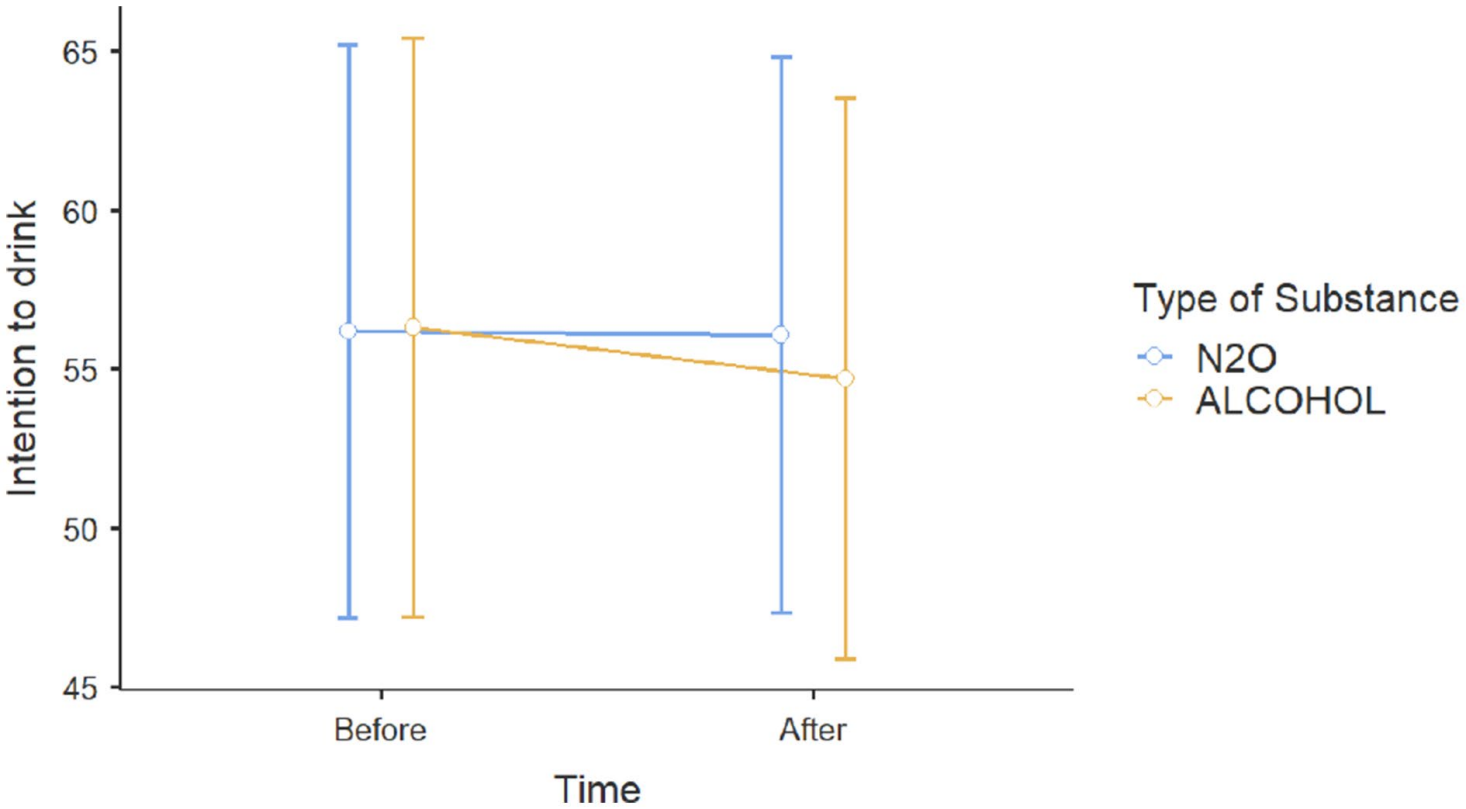
Scores on intention to consume alcohol before and after viewing Instagram posts about either alcohol or N_2_O.

Results for *Action 3* showed on effects on intentions toward alcohol for participants with alcohol prevention posts no age effect [*β* = 0.362, *t*(116) = 1.256, *p* = 0.212, *η*_p_^2^ = 0.013], sex effect [*β* = −5.301, *t*(116) = −1.469, *p* = 0.145, *η*_p_^2^ = 0.018], correct response effect to the alcohol questionnaire [*β* = −0.940, *t*(116) = 0.358, *p* = 0.721, *η*_p_^2^ = 0.001], or exposure type effect (alcohol vs. N_2_O, *p* = 0.618, *η*_p_^2^ = 0.002) on intentions to consume alcohol (Figure 4).

**Figure 5:**
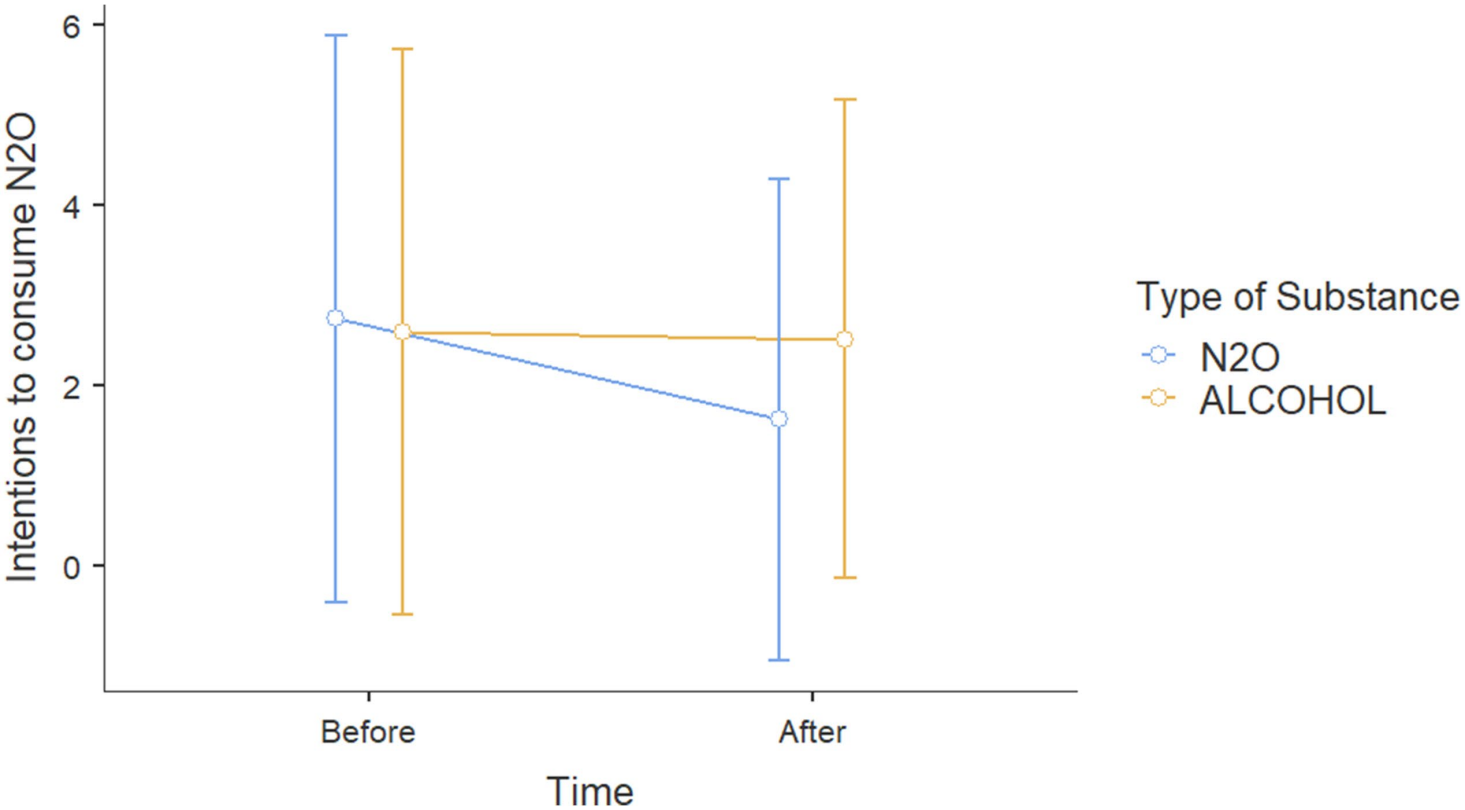
Intention to use N_2_O scores before and after viewing Instagram posts about either alcohol or N_2_O.

Results showed on effects on intentions toward nitrous oxide for participants with nitrous oxide prevention posts no age effect [*β* = −0.403, *t*(64) = −0.278, *p* = 0.782, *η*^2^p = 0.001], no sex effect [*β* = −0.337, *t*(64) = −0.193, *p* = 0.847, *η*_p_^2^ = 0.001], or exposure type effects were found on intentions to consume N_2_O (*p* < 0.17). No significant effect was observed (Figure 5).

**Figure 6:**
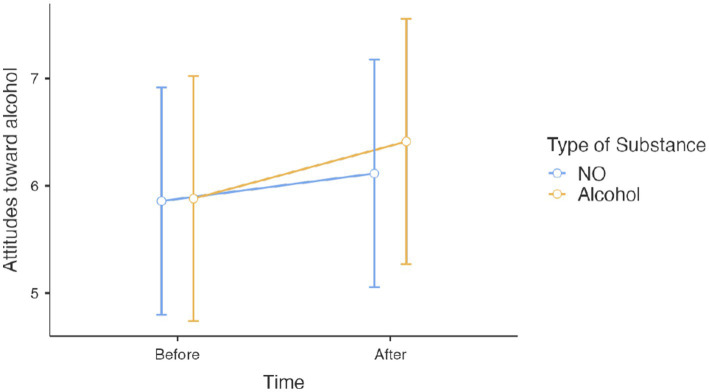
Scores for attitudes to alcohol before and after viewing the website for either alcohol or N_2_O.

**Figure 7:**
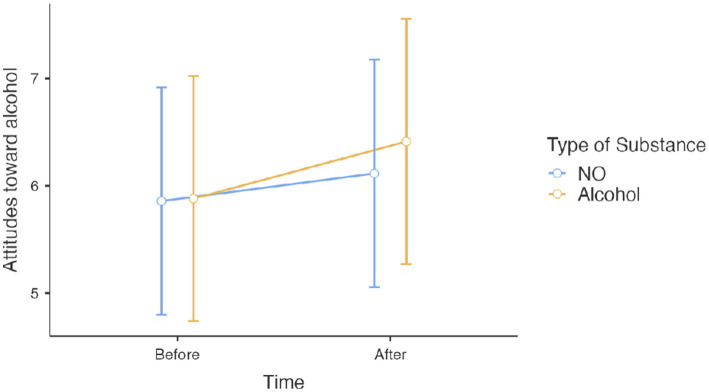
Attitude scores for N_2_O before and after viewing the website for either alcohol or N_2_O.

**Figure 8:**
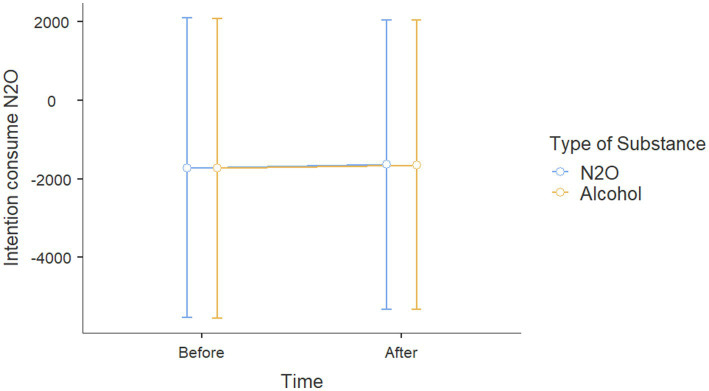
Scores for intentions to consume N_2_O before and after viewing the website for either alcohol or N_2_O.

**Figure 9:**
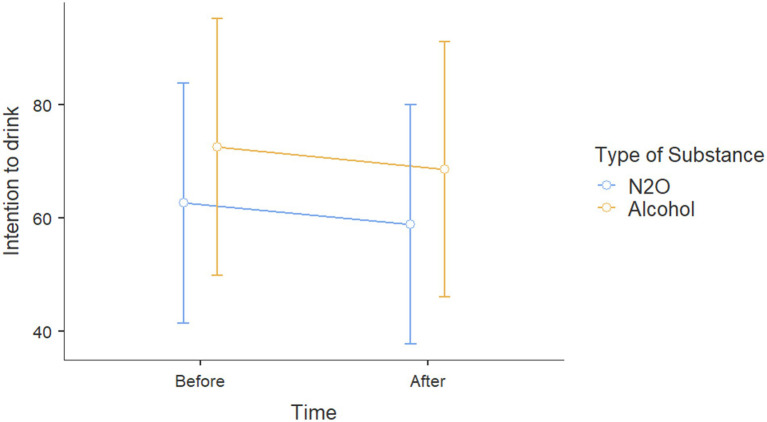
Scores for intentions to consume alcohol before and after viewing the website for either alcohol or N_2_O.

Results for *Action 4* showed that exposure to alcohol prevention content led to more negative attitudes toward alcohol [*F*(1,124) = 3.86, *p* = 0.052, *η*_p_^2^ = 0.03]. The other predictors had no significant effects on attitudes (see Figure 6). We found no significant effect on attitudes toward N_2_O (see Figure 7), on intentions to consume alcohol (*p* > 0.10) (see Figure 8) or on intentions to consume N_2_O (*p* > 0.10) (see Figure 9).

## General discussion

In response to a growing trend in psychoactive substance use among young adults aged 18 to 25, the Avenir Santé Association launched a prevention program specifically targeting ‘emerging substances’, primarily CBD, MDMA, and N_2_O. This multifaceted program had four key parts: (i) training of young volunteers or employees of the association, (ii) direct engagement with young people in popular gathering areas, (iii) use of Instagram for prevention outreach, and (d) awareness raising of party organizers through a dedicated website, montetasoiree.com. The impact evaluation of the program yielded encouraging results. Training participants demonstrated an increased understanding of psychoactive substances, particularly MDMA—a substance that was previously less well understood. This confirmed the effectiveness of the training in increasing knowledge, particularly in areas where knowledge was initially lacking. More precisely, our results showed a general increase in knowledge of all the psychoactive substances studied. An interesting pattern was observed in the change in knowledge from the beginning to the end of the training session: the most pronounced increase in knowledge was for MDMA, followed by CBD and N_2_O. This suggests that the training was particularly effective in promoting understanding of MDMA, possibly owing to the nature of the information presented or the receptiveness of the participants to this particular topic.

Concerning direct action with young people, we found a significant association between knowledge of the health effects of substance use and intention to reduce consumption. This underlines the importance of educational programs that inform potential users about the possible negative health effects of substance use. Age and sex emerged as significant predictors of intentions to reduce substance consumption. Older persons and males were found to have more intention to reduce their consumption of alcohol and tobacco. This could reflect a greater awareness or experience of the negative effects of substance use in older populations (Degenhardt et al., 2007) and males (Becker and Hu, 2008), or it might relate to societal expectations and pressures, which differ by age and sex. Interventions should therefore be tailored to the specific needs and concerns of different demographic groups. We found that exposure to discourse about alcohol during an outreach session had a clear statistically significant effect on participants’ intentions to use alcohol. Discussions about N_2_O showed a marginally significant effect on intentions to use new substances. These findings suggest that what substance is being addressed during outreach initiatives can modulate consumption intentions. However, although the specific topic of the outreach discourse plays a role, the continued importance of intrinsic motivations for substance uses in shaping overall consumption patterns must be emphasized. The findings suggest that interventions aimed at reducing substance use should focus on increasing knowledge about health effects (White et al., 2010), especially for emerging substances. They should also target specific populations across different regions, taking into account regional variations and the demographic factors age and sex (White et al., 2010; Cerdá et al., 2011).

On actions using social networks, exposure to Instagram posts on the effects of addictive substances seemed to affect judgments about these substances. Participants exhibited more negative attitudes toward alcohol when they were exposed to posts about alcohol. Similarly, attitudes toward N_2_O were more negative when participants were exposed to prevention posts about N_2_O and retained information about its effects. This suggests that online prevention campaigns can be effective in changing attitudes and perceptions about substances, which could potentially impact future consumption behaviors. This is in line with previous results observed on a prevention program on Facebook (Flaudias et al., 2015). Despite changing attitudes toward substances, the study found that exposure to the Instagram posts did not significantly affect intentions to consume these substances. Importantly, although attitudes can influence behavior, they do not necessarily translate into actual behavior change. For example, according to the theory of planned behavior (Ajzen, 1991), intention is the most proximal determinant of behavior. However, intention itself is influenced not only by attitudes toward the behavior, but also by several other factors such as subjective norms (perceived social pressure to enact or not enact the behavior) and perceived behavioral control (the extent to which a person feels able to enact the behavior). In our context, it could be that the exposure to Instagram posts primarily affected the attitudes toward substance use, but it might not have significantly influenced subjective norms or perceived behavioral control. For example, although young persons developed negative attitudes toward substances after seeing the Instagram posts, they might still perceive high peer pressure to consume substances (subjective norms), or they might not feel they have much control over their substance use behavior (perceived behavioral control). These factors could counteract their changed attitudes, and result in unchanged intentions to consume substances. Another factor to consider is the gap between intentions and actual behaviors, which can be influenced by a range of factors including forgetting, distraction, and changes in situation or context. That intentions have been affected does not necessarily guarantee a change in actual behavior. The effectiveness of health messages in social media depends on how the messages are designed and presented. If the Instagram posts did not use persuasive techniques effectively (e.g., by not sufficiently tailoring the messages to the target audience, or by not presenting the information in a compelling way), the impact on the viewers’ intentions could be limited. This suggests that whereas the campaign was effective at increasing awareness and changing perceptions, more intensive or different types of intervention might be needed to change consumption intentions and behavior (Wakefield et al., 2010). Interestingly, it was found that the more accurately the participants retained information from the posts about the respective substances (alcohol or N_2_O), the stronger the change in their attitudes. This points to the importance of clear and effective communication in such campaigns. The more successfully the message is delivered and understood, the greater the potential impact on attitudes. Age and sex did not significantly affect attitudes or intentions toward alcohol or N_2_O, showing that the observed effects of the prevention campaign were consistent across different demographic groups in this sample. Regarding intentions to consume emerging substances, we note that participants had extremely low scores to begin with and that these remained very low (about 3% of respondents) throughout the study. This could mean that because the prevention messages were affirming already low intentions to use, little change was elicited.

In conclusion, while the prevention campaign on social network influenced participants’ attitudes toward alcohol and N_2_O, it did not appear to impact their consumption intentions. This suggests that future campaigns may need to couple information dissemination with additional strategies to effectively reduce substance use intentions and behaviors. However, our results highlight the potential power of social media as a platform for health communication and intervention.

Overall, *Action 4* shows that the presentation of information via a website did not have any significant effect on the consumption intentions of alcohol or emerging substances. Nor was there any observed effect on attitudes toward emerging substances. We found no significant change in the participants’ intentions to consume alcohol or N_2_O after exposure to web pages providing information about these substances. This suggests that the website content was not persuasive enough to change behavior intentions or that the participants were already aware of the effects of these substances. Despite exposure to the website’s content about emerging substances, no significant effect was observed on participants’ attitudes toward these substances. One interpretation could be that the participants either already held strong attitudes that were not swayed by the website content, or the content was not engaging or convincing enough to alter the participants’ attitudes. However, the prevention content about alcohol did lead to slightly more negative attitudes toward alcohol although the effect was at the threshold of significance. Hence the website could potentially shape the attitudes of its users toward alcohol consumption, even though it failed to influence their actual intentions to consume alcohol or other substances. The fact that the *Action 4* sample consisted mainly of students rather than party organizers may partly explain these observations. A targeted evaluation involving people interested in organizing parties, such as members of student unions, might therefore yield different findings.

### Limitations

Our evaluation has some limitations. The first action is based on self-observation, and there may be a test-retest effect. Even so, we observed an overall improvement in all items.

Although the results provide an encouraging indication of the effectiveness of the training, the modest sample size limits generalizability. Future research with larger participant pools will help validate these findings and further explore the impact of such training sessions on improving knowledge about psychoactive substances. Subsequent research can also explore the factors that influence different rates of learning for different substances, in order to optimize the design and content of the training.

In *Action 2*, there may have been some selection bias, since the evaluations may have targeted the people who were the most satisfied with the intervention, and therefore probably the most sensitive to the discourse. Further, by asking about behavioral intentions immediately after the intervention, we were not assessing the reflection that the exchange might have elicited, and which could have ultimately modified behavioral intentions. The interviews were also of short duration (13 min, SD = 6 min). *Actions 3 and 4* were carried out with psychology students, a population which, although part of the target population for Avenir Santé Association interventions, may not accurately represent the total population targeted by Avenir Santé Association. It would be of interest to replicate the study controlling for respondents staying the same amount of time on the website, a pitfall that was not present for the Instagram posts. Secondly, it would be necessary to ensure that the amount of information to be retained is the same for both communication modalities. It is possible that the information presented via social networks was (i) less abundant and (ii) presented in a way that makes it easier to retain. *Actions 3 and 4* were carried out with psychology students, a population that, although part of the target population for Avenir Santé Association interventions, may not accurately represent the total population targeted by Avenir Santé Association.

## Conclusion

The program implemented by Avenir Santé Association offers a compelling framework for substance misuse prevention. The program’s efficacy was reflected in the notable proficiency enhancement observed among trained facilitators and an appreciable upsurge in knowledge about emerging substance use trends. Additionally, some individuals demonstrated a promising inclination to reduce their substance consumption. The program’s field interventions fostered an enhanced understanding and amplified aversive attitudes toward a range of substances comprising alcohol, tobacco, CBD, MDMA, and N_2_O among young people. They did not, however, elicit an intent to diminish the consumption of emergent substances, likely owing to the current low usage rate (3%) in the youth population.

The influence of social media as a preventive tool was underlined by a discernable shift in attitudes among young individuals exposed to Instagram posts regarding N_2_O. This underscores the potential of digital platforms for shaping perceptions and catalyzing behavior modification. While these findings contribute significantly to the field, we emphasize that influencing attitudes does not invariably cause behavior change. Future interventions should therefore integrate these insights to frame multifaceted strategies that extend beyond awareness and education, thereby achieving a greater impact on consumption behaviors.

## Data availability statement

The raw data supporting the conclusions of this article will be made available by the authors, without undue reservation.

## Ethics statement

The studies involving humans were approved by Comité d’éthique, de déontologie et d’intégrité scientifique (CEDIS) de Nantes Université (No. IRB: IORG0011023). The studies were conducted in accordance with the local legislation and institutional requirements. The participants provided their written informed consent to participate in this study.

## Author contributions

OZ: Conceptualization, Formal analysis, Methodology, Project administration, Writing – original draft, Writing – review & editing. SL: Conceptualization, Funding acquisition, Investigation, Project administration, Resources, Writing – review & editing. RB: Funding acquisition, Methodology, Project administration, Resources, Writing – review & editing. VF: Conceptualization, Methodology, Project administration, Supervision, Writing – original draft, Writing – review & editing.

## Footnotes

^1^ montetasoiree.com
