# Supplementary material for: Preventing new substance use behaviors in youth: evaluation of a two-year comprehensive program
Source: Front Psychol. 2024 Jul 5;15:1339751. doi: 10.3389/fpsyg.2024.1339751 (PMC11258033; doi:10.3389/fpsyg.2024.1339751)
Supplement: Supplementary file 1 [file Data_Sheet_1.docx]

**APPENDIX A - Questions used to test stakeholders' knowledge of emerging substances in Study 1 (translated from French)**

**CBD**

Question 1: What do you know about CBD?

Question 2: What is the legal status of CBD?

Question 3: What are the different ways it is consumed?

Question 4: What effects do consumers look for?

Question 5: What risk and harm reduction advice could you give?

Question 6: On a scale of 1 to 10, how comfortable do you feel approaching this substance in the field?

**Nitrous oxide**

Question 1: What is nitrous oxide?

Question 2: What effects are consumers looking for?

Question 3: What are the immediate risks of inhaling nitrous oxide?

Question 4: What are the risks of regular use and/or high doses?

Question 5: Is nitrous oxide addictive?

Question 6: What risk and harm reduction advice could you give?

Question 7: On a scale of 1 to 10, how comfortable do you feel about dealing with this substance in the field?

**MDMA**

Question 1: What's the difference between ecstasy and MDMA?

Question 2: What are the different ways it is consumed?

Question 3: What effects do users seek?

Question 4: How can MDMA be detected?

Question 5: To which substance family does MDMA belong?

Question 6: What risk and harm reduction advice could you give?

Question 7: On a scale of 1 to 10, how comfortable do you feel approaching this substance in the field?

**APPENDIX B - Questions used to assess attitudes and intentions to drink and consume emerging substances in Study 2 (translated from French)**

**Nitrous oxide**

1. On a scale of 1 to 10, to what extent do you think the consequences of nitrous oxide consumption could have a negative effect on your health? 1 for no effect, 10 for a significant effect.
2. On a scale of 1 to 10, how much do you intend to reduce your consumption of nitrous oxide in the future? 1 for no intention to reduce, 10 for a strong intention to reduce.
3. On a scale of 1 to 10, how much do you think you intend to continue using nitrous oxide in the future? 1 for no intention to maintain the same consumption, 10 for a strong intention to maintain the same consumption.
4. On a scale of 1 to 10, to what extent do you believe that the consequences of nitrous oxide consumption have no negative effect on your health? 1 for no negative effect, 10 for a significant negative effect.
5. Can you name some places where you can get information, or talk about the substance or your consumption?
6. On a scale of 1 to 10, to what extent do you think the consequences of MDMA use could have a negative effect on your health? 1 for no negative effect, 10 for a significant negative effect.

**MDMA**

1. Have you ever used MDMA in your life?
2. On a scale of 1 to 10, how much do you think you intend to reduce your MDMA consumption in the future? 1 for no intention to decrease, 10 for a strong intention to decrease.
3. On a scale of 1 to 10, how much do you think you intend to continue using MDMA in the future? 1 for no intention to maintain the same level of consumption, 10 for a strong intention to maintain the same level of consumption.
4. On a scale of 1 to 10, to what extent do you feel that the consequences of MDMA use have no negative effect on your health? 1 for no negative effect, 10 for a strong negative effect

**CBD**

1. Have you ever used CBD in your life?
2. On a scale of 1 to 10, to what extent do you think the consequences of CBD consumption could have a negative effect on your health? 1 for no effect, 10 for a significant effect.
3. On a scale of 1 to 10, how much do you think you intend to reduce your CBD consumption in the future? 1 for no intention to decrease, 10 for a strong intention to decrease.
4. On a scale of 1 to 10, how do you rate your desire to continue your CBD consumption in the future? 1 for no intention to maintain the same consumption, 10 for a strong intention to maintain the same consumption.
5. On a scale of 1 to 10, to what extent do you think the consequences of CBD consumption have no negative effect on your health? 1 for no negative effect, 10 for a strong negative effect.
6. Can you name some places where you can get information, or talk about the substance or your consumption?

**Alcohol**

1. Have you ever consumed alcohol in your life?
2. On a scale of 1 to 10, to what extent do you think the consequences of alcohol consumption could have a negative effect on your health? 1 for no effect, 10 for a significant effect.
3. On a scale of 1 to 10, how much do you think you intend to reduce your alcohol consumption in the future? 1 for no intention to reduce, 10 for a strong intention to reduce.
4. On a scale of 1 to 10, how would you rate your intention to continue drinking in the future? 1 for no intention to continue drinking, 10 for a strong intention to continue drinking.
5. On a scale of 1 to 10, to what extent do you believe that the consequences of alcohol consumption have no negative effect on your health? 1 for no negative effect, 10 for a strong negative effect.

Can you name any places where you can get information or talk about the substance or your consumption?

**Tobacco**

1. Have you ever used tobacco in your life?
2. On a scale of 1 to 10, to what extent do you think the consequences of tobacco consumption could have a negative effect on your health? 1 for no effect, 10 for a significant effect.
3. On a scale of 1 to 10, how much do you think you intend to cut down on smoking in the future? 1 for no intention to cut down, 10 for a strong intention to cut down.
4. On a scale of 1 to 10, how much do you think you intend to continue smoking in the future? 1 for no intention to maintain the same level of consumption, 10 for a strong intention to maintain the same level of consumption.
5. On a scale of 1 to 10, to what extent do you feel that the consequences of tobacco consumption have no negative effect on your health? 1 for no negative effect, 10 for a significant negative effect

**APPENDIX C – Instagram Posts and items used in Study 3**

**Posts for nitrous oxide**


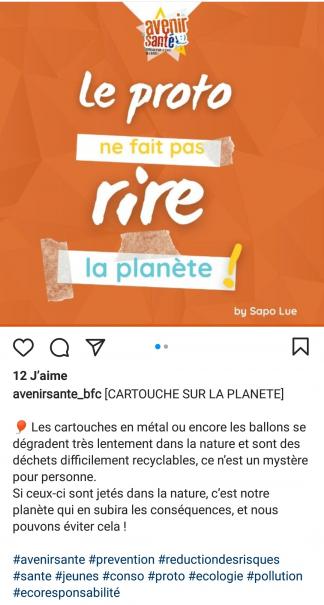

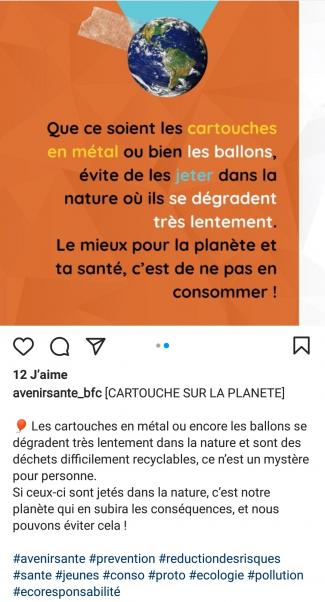
​​​​​​​

Post 1


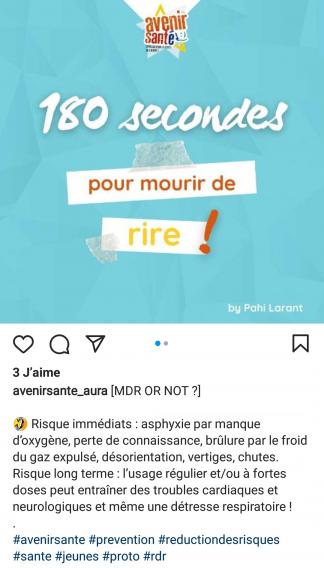

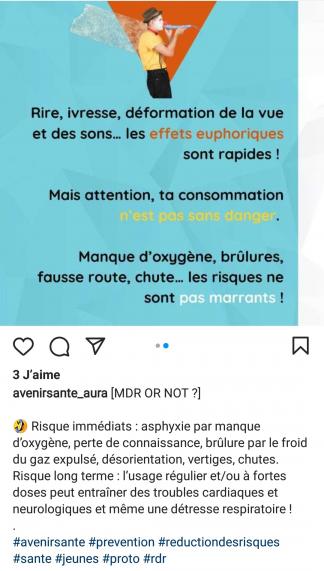


Post 2


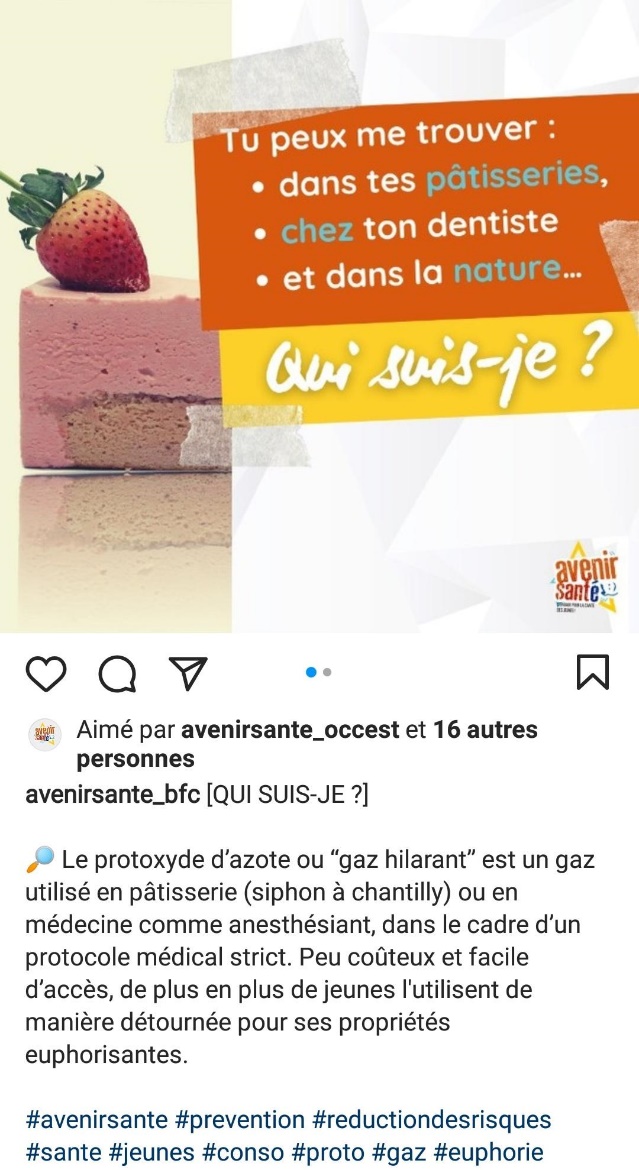

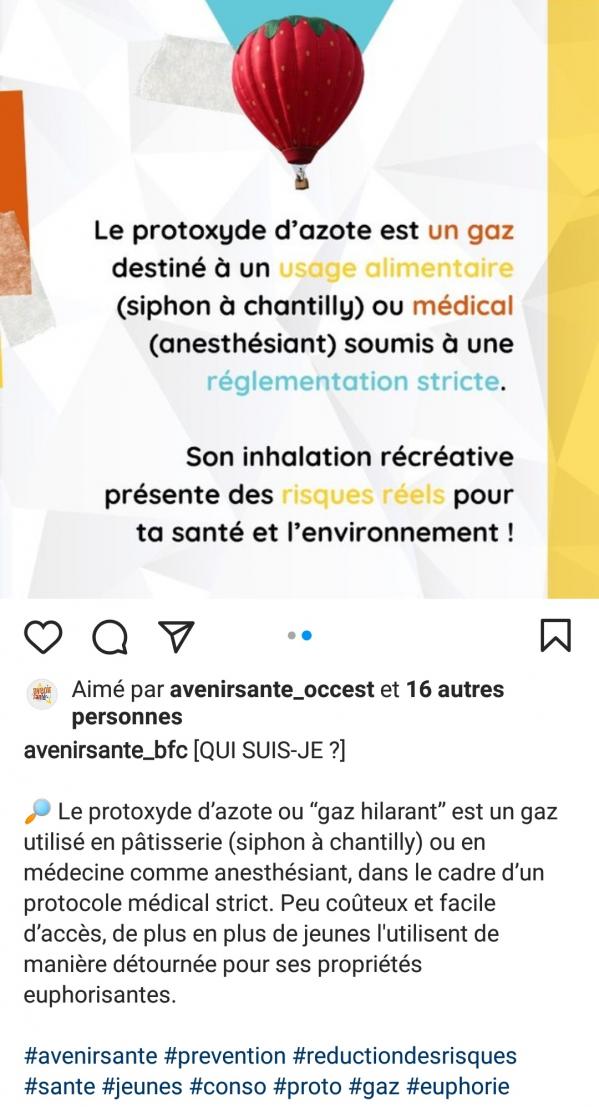


Post 3


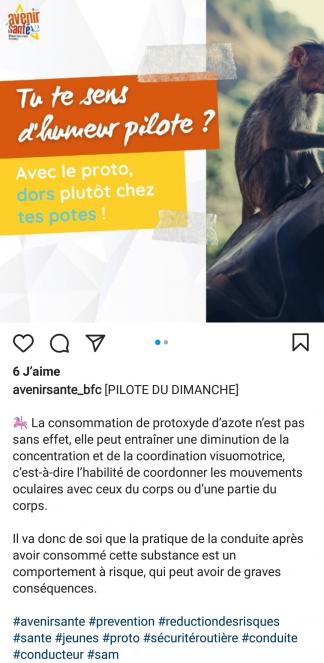

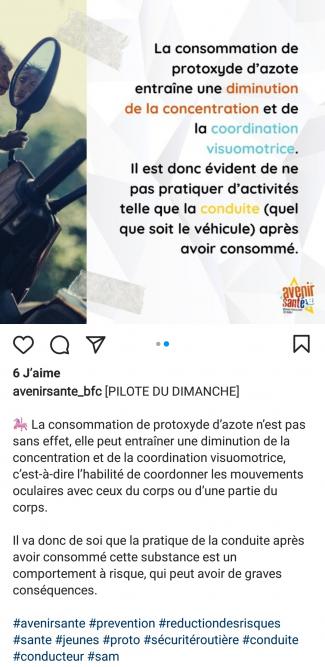


Post 4


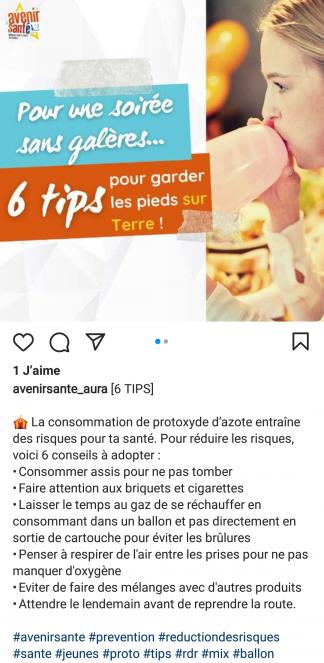

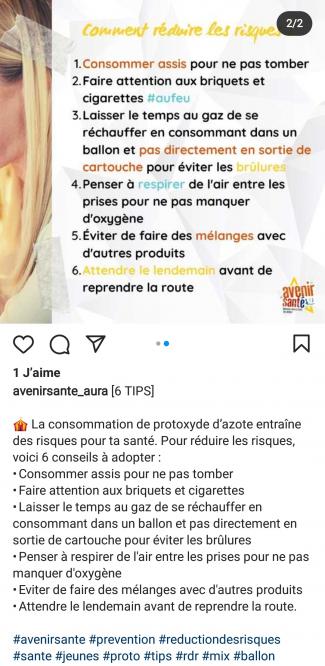


Post 5


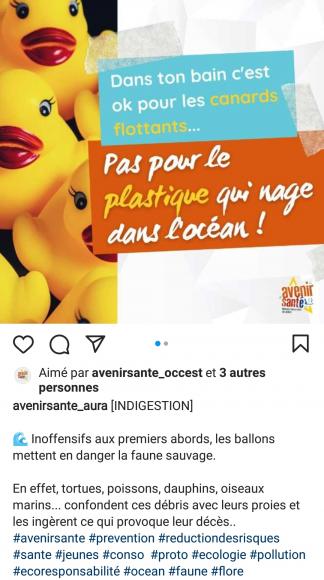
 
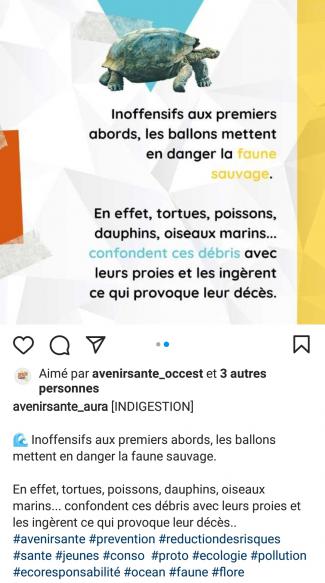


Post 6

**Alcohol**


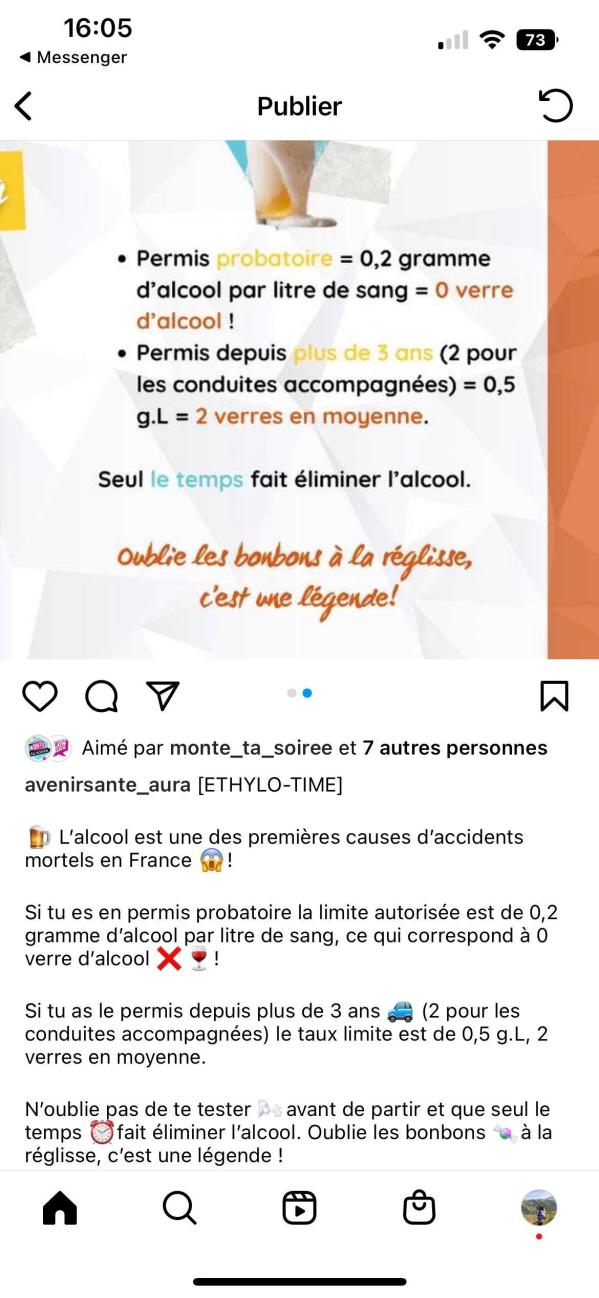
 
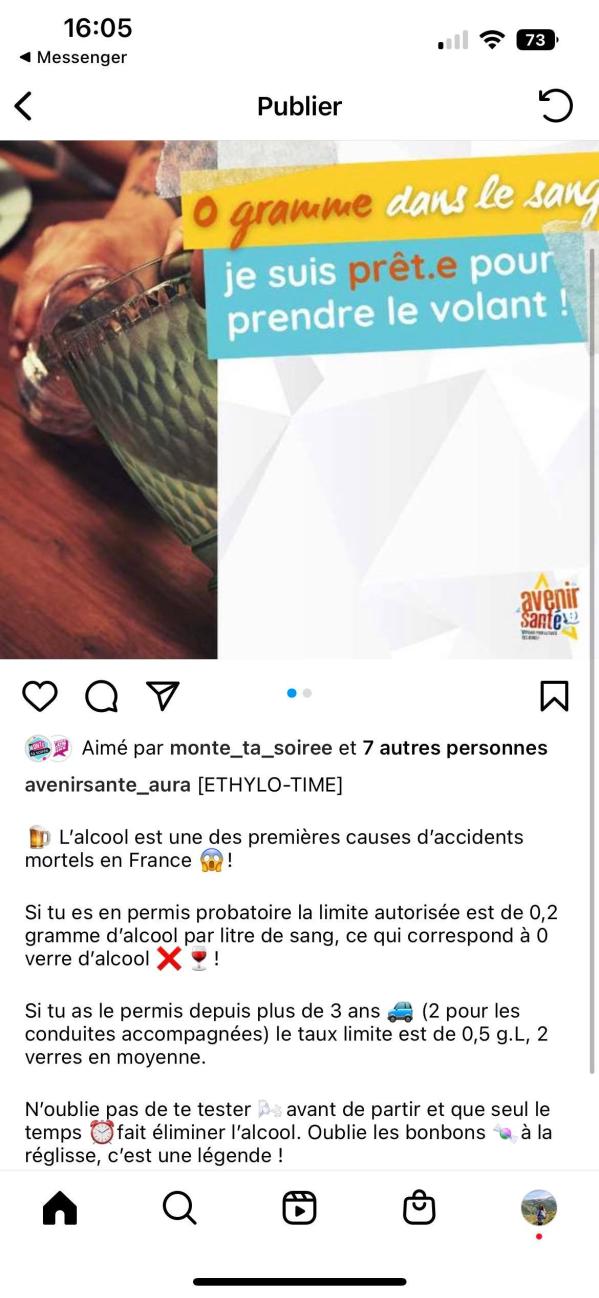


Post 1


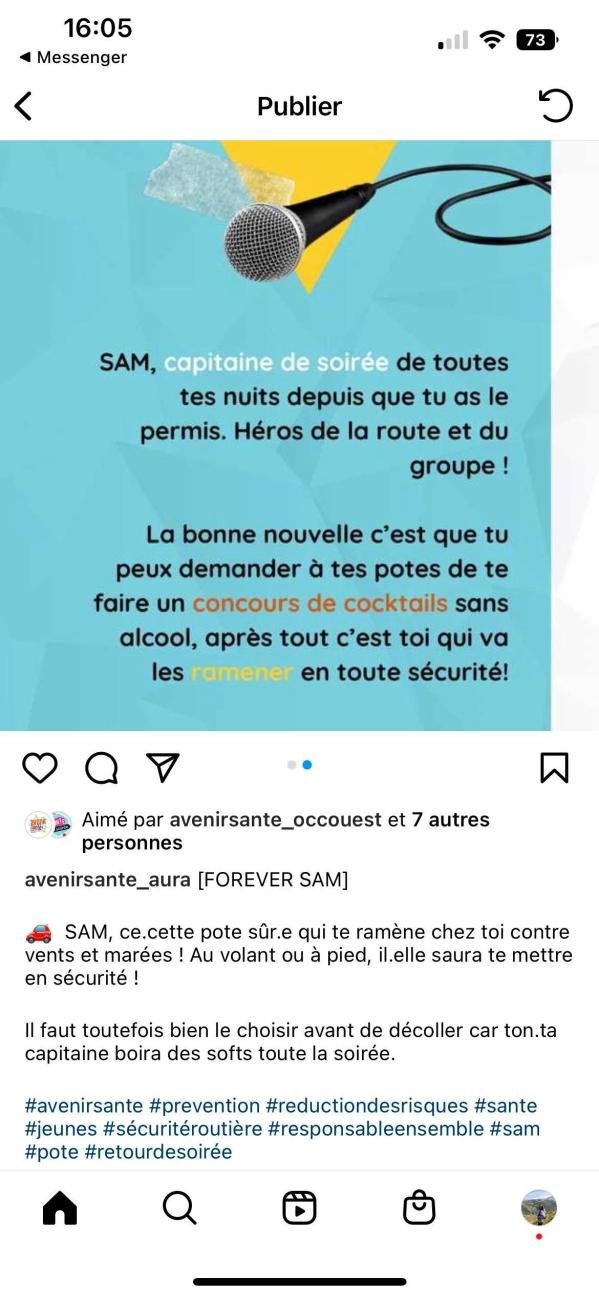
  
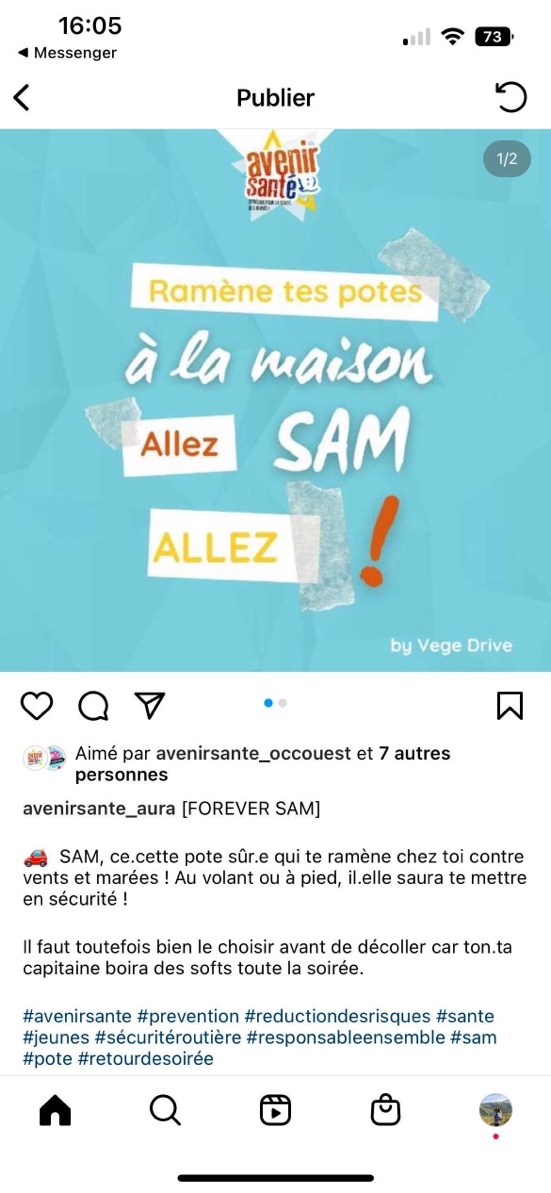


Post 2


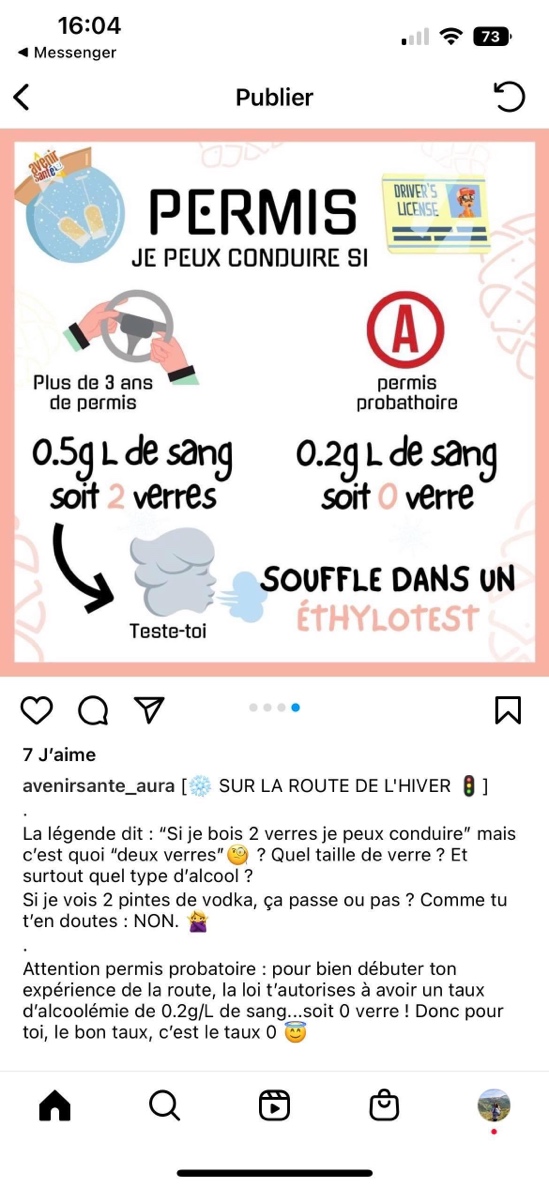

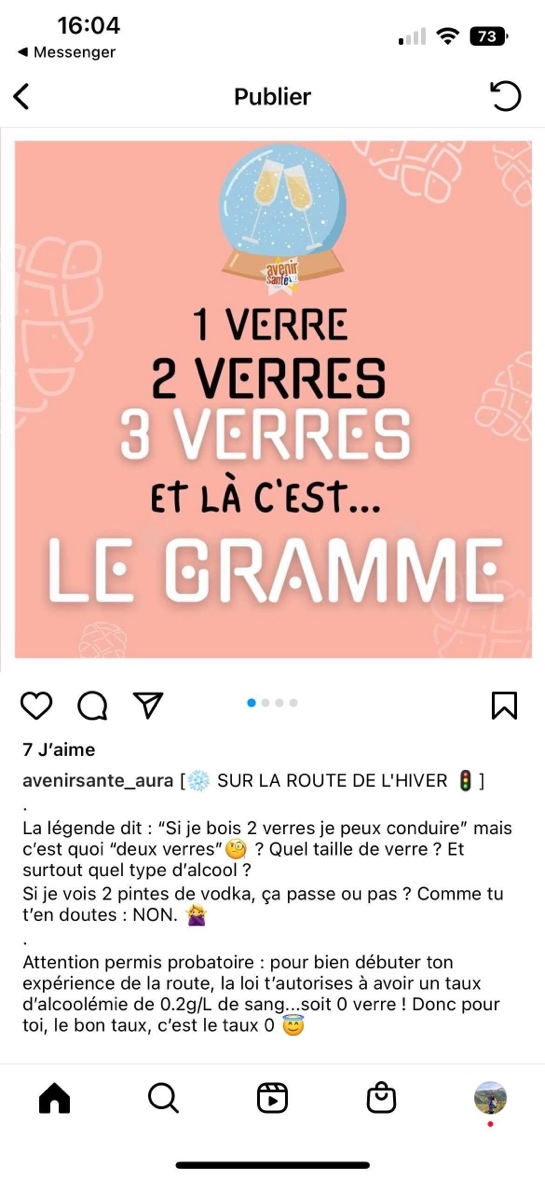


Post 3


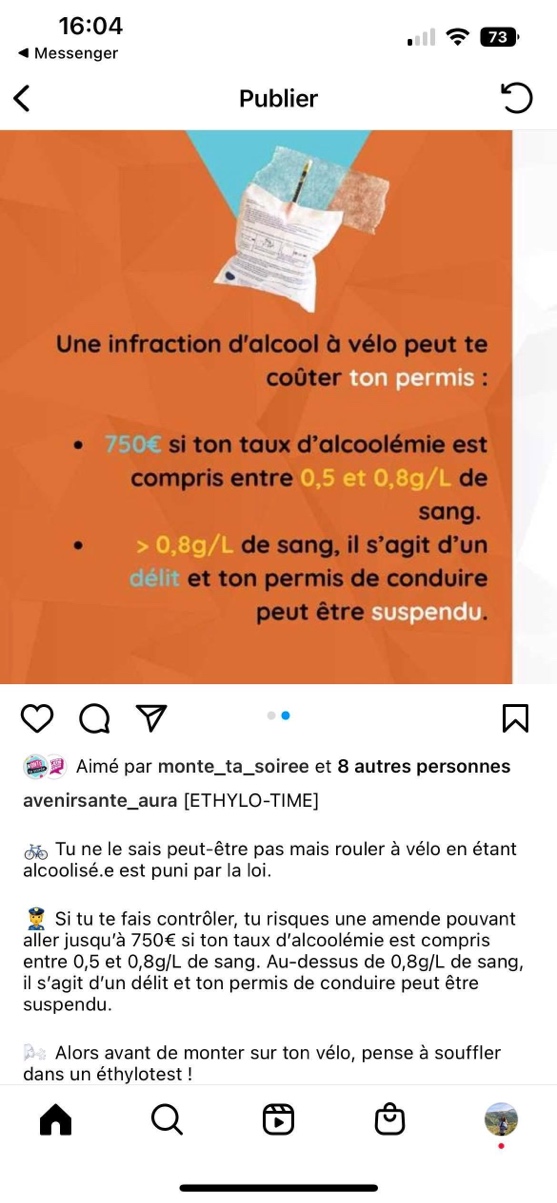

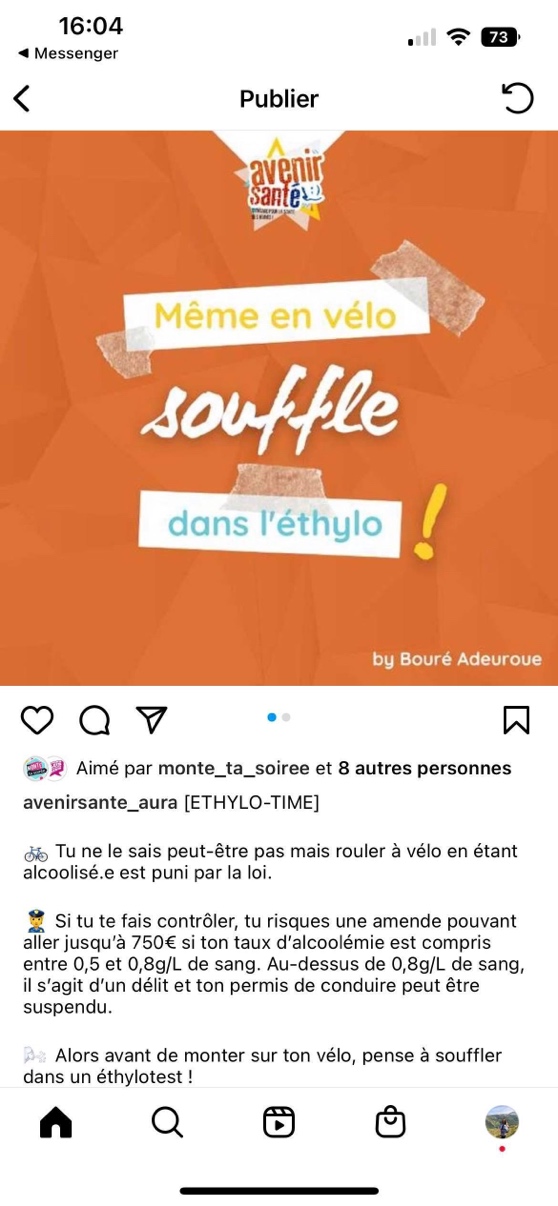


Post 4

 
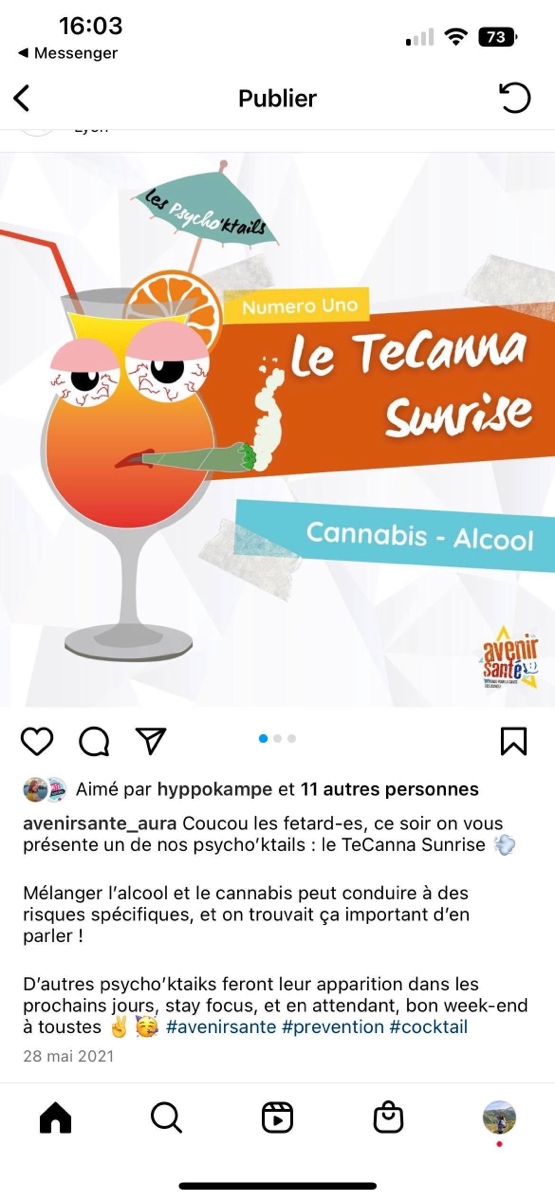
 
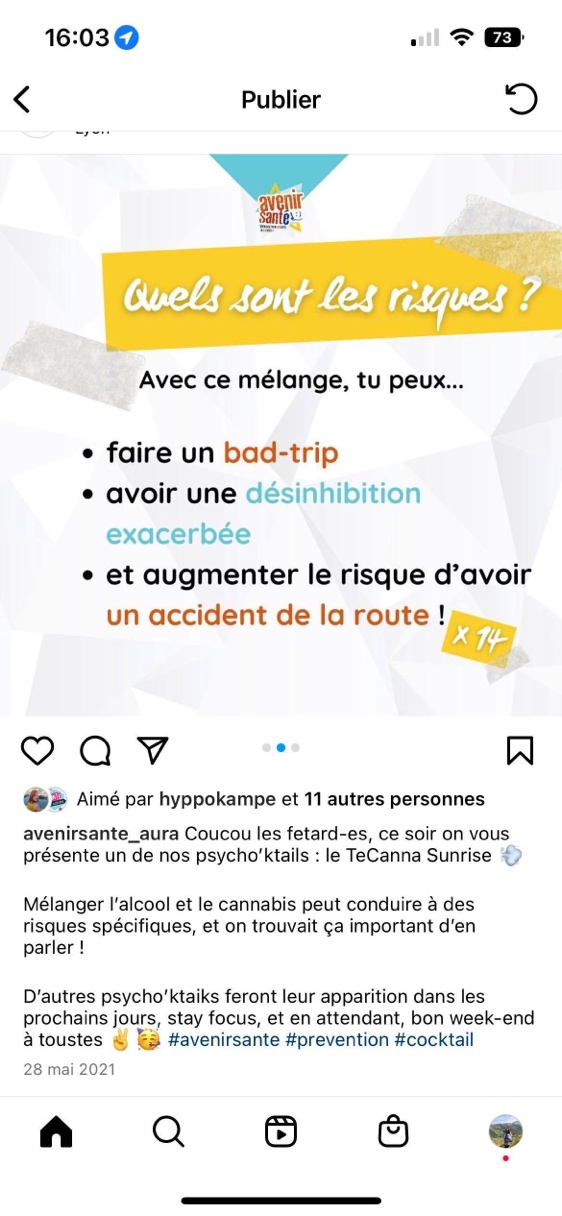


Post 5


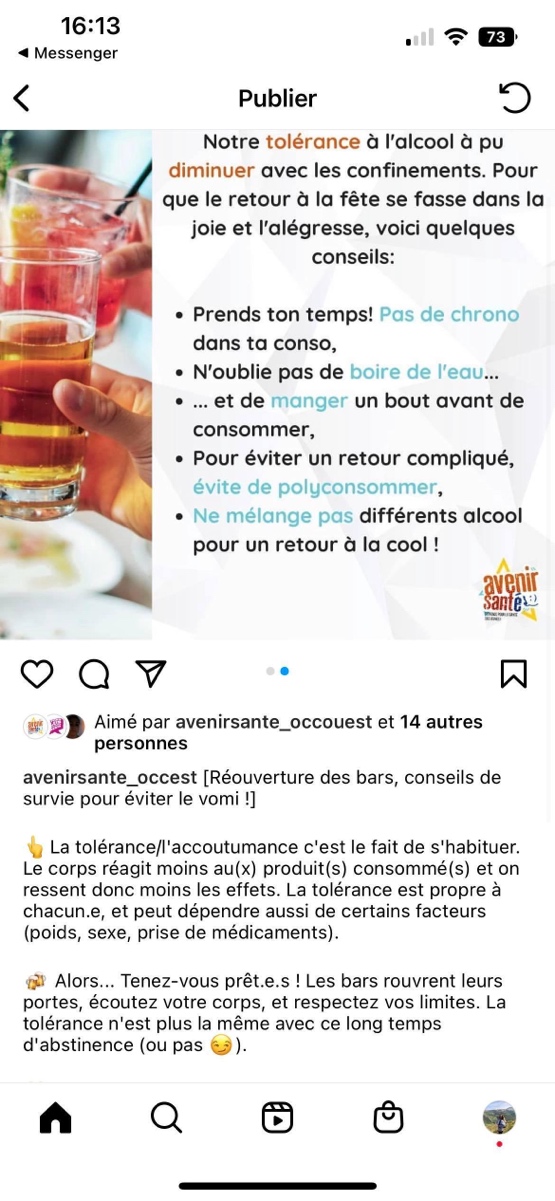

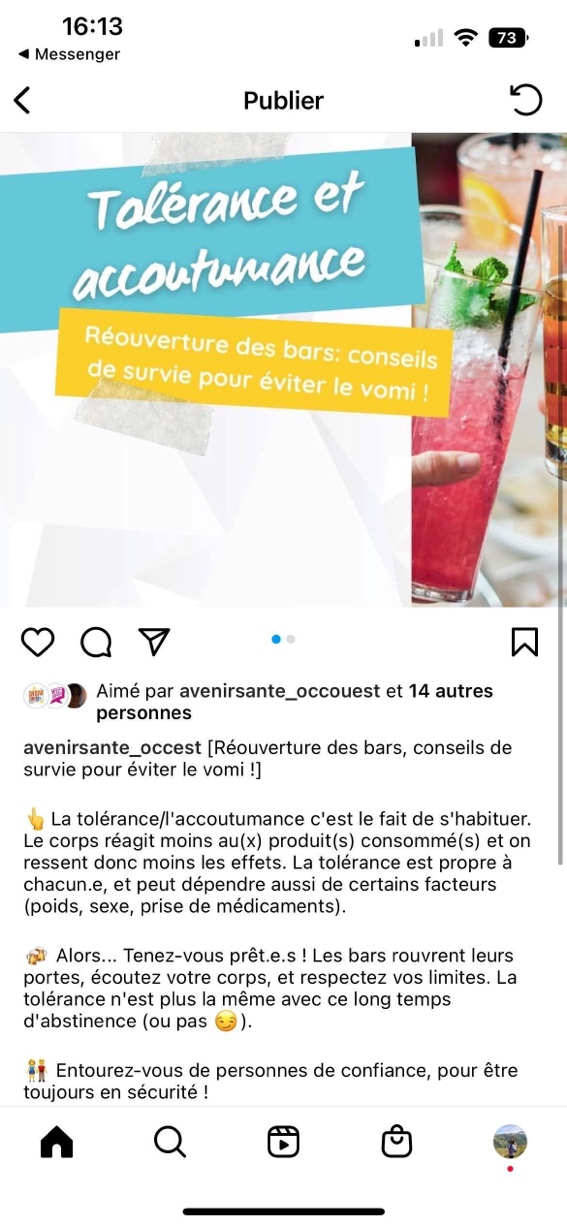


Post 6

**Questions about intentions to drink alcohol/consume nitrous oxide**

1. How much nitrous oxide do you plan to use in the future?
2. How much alcohol do you plan to consume in the future?

**Questions about attitudes toward alcohol and nitrous oxide**

1. Do you think that using nitrous oxide is (i) 1- Healthy to 7- Unhealthy, (ii) 1- Wise to 7- Unwise, (iii) 1- Good to 7- Bad and (iv) 1- Safe to 7- Dangerous?
2. Do you think drinking alcohol is (i) 1- Healthy to 7- Unhealthy, (ii) 1- Wise to 7- Unwise, (iii) 1- Good to 7- Bad and (iv) 1- Safe to 7- Dangerous.

**Questions about memorization**

We're now going to ask you a few questions about the content of the posts you've viewed. Each question may have several correct answers.

What dose of alcohol is equivalent to a glass of wine?
▢ 1 glass of beer (33) 
▢ 1 glass of whisky (33) 
▢ 1 glass of champagne (33) 

To lower blood alcohol levels, you need to: 
▢ Decrease quantities when resuming (25) 
▢ Space out alcohol consumption (25) 
▢ Drink water (25) 
▢ Eat (25) 

Riding a bike while under the influence of alcohol is punishable by law with:
▢ A fine (100) 
▢ Withdrawal of license points (0) 
▢ 3 months' imprisonment (0) 

On a probationary license, what is the authorized alcohol limit?
▢ 0.2 g of alcohol per liter of blood (50) 
▢ 0 glass of alcohol (50) 
▢ 1 glass of alcohol (0) 

If I plan to drive: 
▢ I should think about appointing a party captain (50) 
▢ I shouldn’t drink (50) 
▢ It's okay to drink under 0.5 g of alcohol (3) 

If I mix alcohol and cannabis, I risk: 
▢ Having a road accident (33) 
▢ Being too uninhibited (33) 
▢ Having a bad trip (3) 

What is nitrous oxide?
▢ Medical laughing gas (50) 
▢ Laughing gas for food use (50) 
▢ A toxic gas (0) 

Consuming nitrous oxide:
▢ Increases the probability of having a road accident (100) 
▢ Makes you more alert while driving (0) 
▢ Interacts with alcohol to make you drowsy (0) 

What are the short-term risks of nitrous oxide consumption?
▢ Asphyxia (25) 
▢ Loss of consciousness (25) 
▢ Burns (25) 
▢ Disorientation (25) 

What are the long-term risks of nitrous oxide consumption?
▢ Heart disorders (33) 
▢ Neurological disorders (33) 
▢ Respiratory distress (33) 

Discarded waste nitrous oxide cartridges:
▢ Are a cause of pollution in general for the planet (50) 
▢ Can kill animals that ingest them (50) 
▢ Are not likely to kill animals (0) 

If I’m going to consume nitrous oxide, it's best to do it: 
▢ Sitting (20) 
▢ Standing (0) 
▢ Using a balloon (20) 
▢ Breathing in air between balloons (20) 
▢ Without mixing (20) 
▢ Without driving afterwards (20) 
▢ Without breathing in air between balloons (0) 
